# Nationwide system to centralize decisions around ECMO use for severe COVID-19 pneumonia in Japan (Special Correspondence)

**DOI:** 10.1186/s40560-020-00445-4

**Published:** 2020-04-24

**Authors:** Japan ECMsOnet for COVID-19

**Affiliations:** Japan ECMOnet for COVID-19 Japanese Society of Intensive Care Medicine, the Japanese Association for Acute Medicine, the Japanese Society of Respiratory Care Medicine, and the Japanese Society of PCPS/ECMO, https://jintensivecare.biomedcentral.com/articles/10.1186/s40560-020-00440-9

**Keywords:** Mechanical ventilation, Compliance, Acute respiratory failure, KL-6, Prognosis

## Abstract

The novel coronavirus disease 2019 (COVID-19) is spreading in Japan. We have collected a total of 26 patients with COVID-19 who required extracorporeal membranous oxygenation (ECMO). The available data from the first 14 cases demonstrated that the median age of patients was 71 and the median PaO_2_/FIO_2_ ratio, positive end-expiratory pressure, mean airway pressure, and lung compliance were 70, 15 cmH_2_O, 21 cmH_2_O, and 28 mL/cmH_2_O, respectively. Median serum KL-6 level was 333 U/mL. Consequently, 16 (62%) out of the 26 have been weaned off and 6 (26%) have been extubated and on rehabilitation, while the other 10 (38%) remain on ECMO. There seemed to be two phenotypes of COVID-19: one with impaired lung compliance and one with preserved lung compliance. The latter phenotype was likely to be favored from the use of ECMO. Further investigation is necessary to clasrify the optimal use of ECMO in patients with COVID-19.

## Main Text

The novel coronavirus disease 2019 (COVID-19) is spreading in Japan. The number of patients who need extracorporeal membranous oxygenation (ECMO) is expected to increase; however, the clinical characteristics of the patients who require and will benefit from ECMO are unclear [1].

On February 15, the Japanese Society of Intensive Care Medicine (JSICM), the Japanese Association for Acute Medicine (JAAM), the Japanese Society of Respiratory Care Medicine (JSRCM), and the Japanese Society of PCPS/ECMO (JSPCPS/ECMO) launched “Japan ECMOnet for COVID-19” as a telephone consultation, treatment support, and a web-based real-time nationwide registry and surveillance system to discuss COVID-19 patients from over 400 hospitals who may be candidates for ECMO [2]. The initiative is led by more than 20 ECMO experts (Japan ECMOnet for COVID-19) from all over Japan.

As of March 15, there have been 26 patients who were placed on ECMO based on deliberation of the group. Sixteen out of the 26 (61.5%) have been weaned off and six have been extubated and on rehabilitation, while the rest remain on ECMO. A few of these patients who have been weaned off ECMO still need treatment for other organ failures.

We report the data from the first 14 cases. The median age of the patients is 71 (range 45–81 years). The median number of days between intubation and ECMO was 3 days (range 0–9 days). The median PaO_2_/FIO_2_ ratio, PEEP, mean airway pressure, and lung compliance before initiation of ECMO were 70 (range 52–147), 15 cmH_2_O (range 10–18 cmH_2_O), 21 cmH_2_O (18–27 cmH_2_O), and 28 mL/cmH_2_O (range 13.6–70 mL/cmH_2_O), respectively. Selected laboratory data of the patients on admission were as follows: median serum KL-6 (a marker of interstitial pneumonia) was 333 U/mL, LDH was 460 IU/L, and procalcitonin was 0.12 ng/mL. With regard to ECMO settings, the median blood flow was 4 L/min (range 2.5–5.3 L/min), the median size of the draining cannula was 24 Fr (range 21–25 Fr), and the median size of the infusing cannula was 20 Fr (range 16–21 Fr). For anti-viral treatment, lopinavir was used for 13/14 (93%) patients. All patients received empirical antibiotics (carbapenems or 3rd/4th generation cephalosporins). Ciclesonide, a glucocorticoid inhaler, was used for 4/13 (31%) of the cases. The effectiveness of any of the medications cannot be assessed at this time.

Experts within Japan ECMOnet for COVID-19 identified two phenotypes of patients with severe pneumonia: one associated with low lung compliance and another with preserved lung compliance. Oxygenation of patients with preserved lung compliance did not improve with higher PEEP. For these patients, the serum KL-6, SP-D (another marker of interstitial pneumonia), and LDH were not elevated on admission.

These findings suggest that lung fibrosis was not severe for this subgroup of patients. The adoption of a platform for real-time discussion to guide the use of a scarce resource such as ECMO has been valuable to the Japanese doctors who are caring for critically ill patients with COVID-19 infection. A central near-real-time data repository is optimal to perform just-in-time epidemiologic studies and to develop algorithms that can inform clinical decision-making.

## Data Availability

Not applicable.

## Abbreviations

COVID-19: Coronavirus disease 2019
ECMO: Extracorporeal membranous oxygenation
FIO_2_: Fraction of inspired oxygen
JAAM: The Japanese Association for Acute Medicine
JSRCM: The Japanese Society of Respiratory Care Medicine
JSICM: The Japanese Society of Intensive Care Medicine
LDH: Lactate dehydrogenase
PaO_2_: Partial pressure of arterial oxygen
PCPS: Percutaneous cardiopulmonary support
PEEP: Positive end-expiratory pressure
SP-D: Surfactant protein-D
