# Correction to: Nationwide system to centralize decisions around ECMO use for severe COVID-19 pneumonia in Japan (Special Correspondence)

**DOI:** 10.1186/s40560-020-00454-3

**Published:** 2020-05-25

**Authors:** Japan ECMOnet for COVID-19

**Affiliations:** Japan ECMOnet for COVID-19 Japanese Society of Intensive Care Medicine, the Japanese Association for Acute Medicine, the Japanese Society of Respiratory Care Medicine, and the Japanese Society of PCPS/ECMO, https://jintensivecare.biomedcentral.com/articles/10.1186/s40560-020-00440-9

**Correction to: J Intensive Care (2020) 8:29**

**https://doi.org/10.1186/s40560-020-00445-4**

In the original publication of this article [1], an error was found in the collaborator name Japan ECMOnet for COVID-19.

The incorrect author name is: Japan ECMsOnet for COVID-19.

The correct author name is: Japan ECMOnet for COVID-19.
